# Gene Expression Analysis of (Paired) Primary and Relapsed Wilms Tumor Samples to Unravel the Underlying Factors Driving Tumor Recurrence

**DOI:** 10.1002/cam4.70969

**Published:** 2025-05-29

**Authors:** Alissa Groenendijk, Jarno Drost, Annelies M. C. Mavinkurve‐Groothuis, Martine van Grotel, Geert O. Janssens, Annemieke S. Littooij, Alida F. W. van der Steeg, Marry M. van den Heuvel‐Eibrink, Lennart Kester, Ronald R. de Krijger

**Affiliations:** ^1^ Princess Máxima Center for Pediatric Oncology Utrecht the Netherlands; ^2^ Oncode Institute Utrecht the Netherlands; ^3^ Department of Radiation Oncology University Medical Center Utrecht Utrecht the Netherlands; ^4^ Department of Radiology University Medical Center Utrecht Utrecht the Netherlands; ^5^ Division of Childhealth, Wilhelmina Children's Hospital University of Utrecht Utrecht the Netherlands; ^6^ Department of Pathology University Medical Center Utrecht Utrecht the Netherlands

## Abstract

**Purpose:**

We aimed to unravel underlying factors driving Wilms tumor (WT) recurrence and to build a prediction model for recurrence based on gene expression data of (paired) primary and relapsed WT samples.

**Experimental Design:**

Gene expression levels from seven paired primary and relapsed WT samples from patients treated in the Princess Máxima Center were compared among each other, as well as to matched primary WT samples of patients without recurrence (controls). The differential gene expression analysis results were run through ToppGene for functional enrichment. We built a 10‐fold ridge regression model to predict relapse based on gene expression levels of the seven primary cases and all other available primary WT controls (*n* = 42).

**Results:**

The comparison of primary WT and paired relapses showed downregulation of genes involved in immune regulation among relapses and upregulation of cancer stem cell (CSC) regulation genes. Comparing these primary WT samples to matched controls, we observed that downregulated genes in primary samples of relapsed patients were related to stromal cells and muscle development, and upregulated genes were associated with CSCs. The prediction model revealed a sensitivity of 57.14% (95% CI: 14.29%–85.71%) and a specificity of 92.86% (95% CI: 83.33%–100%) when predicting WT relapse.

**Conclusion:**

The CSC pool could play a role in relapse through immune regulation and tumor propagation. Differentiation of CSCs into mesenchymal cells might attenuate the risk of relapse. Our prediction model might aid in selecting patients with an increased risk of relapse at primary diagnosis when externally validated.

## 1 Introduction

In high‐income countries, the overall survival (OS) rate for patients with Wilms tumor (WT) has improved to more than 90% with recent treatment protocols [1]. However, approximately 15% of patients experience recurrent disease [2]. Which patients may relapse remains elusive. The prognostic factors currently used for risk stratification in the international WT treatment protocols (advanced tumor stage, high‐risk histology, and combined loss of heterozygosity at 1p and 16q in chemotherapy‐naïve WTs) are present in only one third of patients who relapse [3, 4, 5, 6]. In other words, it remains challenging to predict who might suffer from recurrent disease. This is especially the case among patients with low and intermediate‐risk WT as approximately 40% of all relapses occur in this group [2, 7, 8].

So far, only limited studies have focused on the underlying molecular mechanism for tumor relapse, which are more extensively referred to in the discussion [9, 10, 11, 12]. Briefly, these studies have described chromosomal abnormalities at 1q, chromosome 3, 16q, and mutations in *SIX1/SIX2*, the MYCN network, and microRNA processing genes, including *DROSHA*, as potential driving events in WT relapse (see Table 1). The latest paired samples analysis by Ciceri et al. excluded samples with known *SIX1/SIX2* mutations. Mutations that were maintained between primary and paired relapse samples were mostly involved in DNA damage prevention and repair or chromatin modification and regulation [13].

**TABLE 1:** Published literature on the molecular analysis of paired primary and relapsed Wilms tumor.

Authors (year of publication)	Number of cases	Methods	Reference
Spreafico et al. (2016)	Eight primary and paired relapsed samples	CNV assessment and gene panel mutation analysis: *WT1, WTX, CTNNB1, TP53, SIX1, SIX2, DROSHA, DICER1, DGCR8*	[9]
Ciceri et al. (2021)	Eight paired samples previously described by Spreafico et al.	Gene panel mutation analysis: *SIX1, SIX2, DGCR8, DROSHA, DICER1*	[10]
	Nineteen new paired samples		
	Ten relapse samples (unpaired)		
Gadd et al. (2022)	Forty‐five paired samples	Whole genome sequencing and RNA sequencing^a^	[11]
	Nine relapse samples (unpaired)		
Natrajan et al. (2007)	Ten paired samples	Breakthrough Breast Cancer human comparative genomic hybridization (CNV assessment) and *WT1* mutation analysis	[12]
	Ten relapse samples (unpaired)		
	Twenty‐eight primary tumors known to subsequently relapse (unpaired)		
Ciceri et al. (2024)	Thirteen paired samples previously described by Ciceri et al. (2021)	All samples known to harbor *SIX1/SIX2* mutations were excluded. Gene panel mutation analysis was performed including ~5000 genes	[13]
	Five paired samples previously described by Spreafico et al.		
	Six new paired samples and 4 new relapse		

Abbreviation: CNV, copy number variation.

^a^ RNA sequencing results on transcriptome‐wide comparison are not reported.

These previous studies have focused primarily on the relevance of mutational status and copy number variations (CNVs) regarding WT relapse. Paired gene expression of primary diagnosis and relapse samples, however, has not been studied in detail in this context. To address this gap, we firstly aimed to identify drivers of tumor relapse by comparing expression profiles of primary WTs to paired relapse samples of patients treated in the Princess Máxima Center. Secondly, we aimed to determine gene expression profiles associated with the risk of relapse by comparing expression data of these primary tumors (from patients with subsequent relapse) to the data of matched control WTs (tumors of patients without a relapse and at least 2 years post diagnosis). Finally, we aimed to build a relapse prediction model based on the gene expression data of the primary tumors and all other (unmatched) control samples available in the Princess Máxima Center.

## 2 Methods

### 2.1 Patients

All patients with relapsed WT who were treated in the Princess Máxima Center and had given informed consent were identified from the clinical records. For seven patients, frozen tumor tissue was available for both the primary and corresponding relapsed WT. These patients were included in our study. The patients with a subsequent relapse are further referred to as cases. WT patients diagnosed in the Princess Máxima Center without relapse at least 2 years after the end of treatment served as controls. In addition, these control patients were matched to the patients with relapse based on sex, age at diagnosis, and stage and histology of the primary tumor, if possible. The patients who did not develop subsequent relapse are referred to as controls. All patients were diagnosed between 2017 and 2023, and treated according to the 2001 or 2016 UMBRELLA protocol of the Renal Tumor Study Group (RTSG) of the International Society for Pediatric Oncology (SIOP), i.e., pre‐operative chemotherapy with vincristine and actinomycin‐D in the case of local disease, and additional doxorubicin in the case of metastases or extensive tumor thrombus at diagnosis. Post‐operatively, chemotherapy with vincristine and actinomycin‐D or additional doxorubicin and/or radiotherapy was continued according to the UMBRELLA protocol, or therapy intensity was further increased to include cyclophosphamide, carboplatin, etoposide, and doxorubicin with or without radiotherapy depending on the response to pre‐operative chemotherapy, the histology, and the volume of the primary tumor [1]. No further therapy was given before relapse biopsies were obtained.

### 2.2 DNA and RNA Sequencing

DNA was isolated from the primary and relapsed tumor samples, as well as from blood from the corresponding patients using the AllPrep DNA/RNA/Protein Mini kit according to standard protocol on the QiaCube (Qiagen, Germantown, Maryland). Whole genome/exome sequencing (WGS/WXS) libraries were generated from 150 ng DNA using the KAPA DNA HyperPlus kit (Roche, Basel, Switzerland). The NovaSeq 6000 platform (Illumina, San Diego, California) was used for sequencing (2 × 150 bp). Similarly, RNA was isolated from tumor samples using the AllPrep DNA/RNA/Protein Mini Kit (Qiagen). RNA‐sequencing (RNA‐seq) libraries were generated from 300 ng RNA using the KAPA RNA HyperPrep Kit with RiboErase (Roche) and sequenced with NovaSeq 6000 (2 × 150 bp) (Illumina), aiming for 40 million unique reads.

### 2.3 Data Pre‐Processing

WXS was performed on all samples that underwent sequencing in a diagnostic setting, which is standard practice in the Princess Máxima Center. WGS was performed in patients who had given consent for sequencing in a research setting. WGS, WXS, and RNA‐seq data were processed in accordance with a standardized pipeline implemented in the Princess Máxima Center. This pipeline has previously been described in detail [14]. In brief, sequencing data was analyzed in accordance with the Genome Analysis Toolkit (GATK) best practice guidelines. WGS/WXS data were analyzed to detect single/multi‐nucleotide variants (SNV/MNV) and CNVs, using the GRCh38 as a reference genome. Variants were identified using Mutect2 from GATK by comparing normal (blood) and tumor DNA. Variants were filtered and annotated using Alissa Interpret (Agilent Technologies). For RNA‐seq, the reads were mapped to GRCh38 using STAR aligner [15]. Raw read counts were obtained via featurecounts. STAR‐Fusion was used for gene fusion detection [16]. In both WGS/WXS and RNA‐seq analysis, quality control was performed using Fastqc and PicardTools for detection of duplicate reads.

### 2.4 Transcriptome Analysis

Raw read counts from RNA‐seq were normalized using the VST normalization function of R package DESeq2 (version 1.40.2) [17]. Principal component analysis (PCA) was performed using the pcaPlot function (DESeq2). The genes contributing to the different PC's were identified using the factoextra package in R (version 1.0.7) [18]. Differential gene expression was performed using the DESeq2 pipeline with a cut‐off of adjusted *p*‐value < 0.05. Gene list enrichment analysis on the gene sets that were significantly upregulated and downregulated was performed using the ToppGene suite [19].

### 2.5 Immune Cell Deconvolution

Immune cell composition of all samples described in the manuscript was estimated using CIBERSORTx [20]. Gene expression data (in CPM format) was uploaded to the CIBERSORTx web platform (available via: https://cibersortx.stanford.edu/). The LM22 signature matrix, as defined by CIBERSORTx, was applied. We ran the analysis with Batch correction (B‐mode) and disabled quantile normalization. The immune cell proportions were visualized using R package ggplot2 (version 3.5.1) [21] and statistically compared using the Wilcoxon signed‐rank test to compare paired samples and the Mann–Whitney *U* test to compare independent samples from the R package ggpubr (version 0.6.0) [22].

### 2.6 Relapse Prediction Model

To build a ridge regression model for the prediction of WT relapse we used gene expression data of primary WTs with subsequent relapse and of all primary WTs without subsequent relapse (and at least 2 years of follow‐up) with available RNA‐seq data collected in the Princess Máxima Center. The patients without subsequent relapse served as a control cohort (*n* = 42). The model was built using a 10‐fold cross‐validation approach (given the relatively low number of primary samples with subsequent relapse). A model fit was computed within each of the folds. For each fold, DESeq2 was applied to determine the differentially expressed genes between the primary WT cases with subsequent relapse and the controls, a binomial ridge regression model was created using the glmnet R package (version 4.1‐8) [23], with alpha set to 0. The model was used to predict the number of recurrences in each fold that was left out. Receiver operating characteristic (ROC) curves were plotted using the pROC package in R with default settings (version 1.18.4) [24]. Patient characteristics of true positives and false negatives were compared using R package finalfit (version 1.0.6) [25], including chi‐square analysis for categorical variables and student t‐test for continuous variables. We aimed to validate the model on the TARGET WT dataset (publicly available through Genomic Data Commons (https://portal.gdc.cancer.gov/)). Validation was done as follows. Gene expression counts were downloaded through the gdc data portal. Expression counts were normalized to counts per million. We then used the 10 models constructed in the cross‐validation setting on our own cohort to predict the risk of recurrence for each of the samples in the TARGET cohort. Risk predictions were averaged across the 10 models and the average predicted risk was used in a Cox proportional hazard model to test whether the predicted risk was significantly associated with the time to relapse/progression.

## 3 Results

### 3.1 Clinical Samples

We identified seven patients with a WT and subsequent relapsed disease with available frozen tumor tissue and/or WXS/WGS and RNA‐seq data. Patient, tumor, and first line treatment characteristics are described in Table 2. The majority of patients (6/7) were male, median age at diagnosis was 77 months, and tumor histology of most primary WTs was intermediate risk (6/7). Only one patient presented with high‐risk, blastemal‐type primary WT. One patient presented with a tumor thrombus reaching the right atrium. Another two patients presented with lung metastases at diagnosis. Most relapses (5/7) occurred in the lung. One of the patients with lung metastases at diagnosis also relapsed in the lung. The characteristics of the control patients (patients without relapse) that were matched to the seven patients with relapse are presented in Table S1. As can be observed, the average age of the control patients is lower, which is due to the fact that priority was given to match on stage and histology at primary diagnosis, rather than sex and age.

**TABLE 2:** Characteristics of study patients.

Patient	Sex	Age at diagnosis (months)	Tumor stage	Tumor histology (primary)	Risk group	First line pre‐op therapy	First line post‐op therapy	Relapse location	Tumor histology (relapse)^a^	RFS (months)	Follow‐up since end of first line treatment (months)
1	M	66	I	Blastemal	High	VA	VAD	Lung	75% B	33	80
									25% E		
2	F	53	III	Mixed	Intermediate	VAD	VA + RT	Retroperitoneum	100% B	9	55
3	M	167	III	Mixed	Intermediate	VA	VA + RT	Lung	75% B	42	75
									20% E		
4	M	40	II	Mixed	Intermediate	VA	VA	Lung	80% B	10	40
									15% S		
5	M	177	III	Mixed	Intermediate	VA	VAD + RT	Lung	50% B	12	28
									30% E		
									20% S		
6	M	77	II (IV)	Mixed	Intermediate	VAD	CyCED + RT	Lung	50% B	39	54
									50% E		
7	M	105	III (IV)	Regressive	Intermediate	VAD	CyCED + RT	Liver	70% B	33	42
									30% E		

Abbreviations: B, blastema; CyCED, cyclophosphamide, carboplatin, etoposide, and doxorubicin; E, epithelium; F, female; M, male; RFS, relapse‐free survival; RT, radiotherapy; S, stroma; VA, vincristine, and actinomycin‐D; VAD, vincristine, actinomycin‐D, and doxorubicin.

^a^ No official histological classification of the International Society of Pediatric Oncology–Renal Tumor Study Group (SIOP–RTSG).

### 3.2 Identifying Drivers of Relapse: Comparing Paired Primary and Relapsed WT Samples

#### 3.2.1 Genetic Aberrations in Paired Samples

**FIGURE 1:**
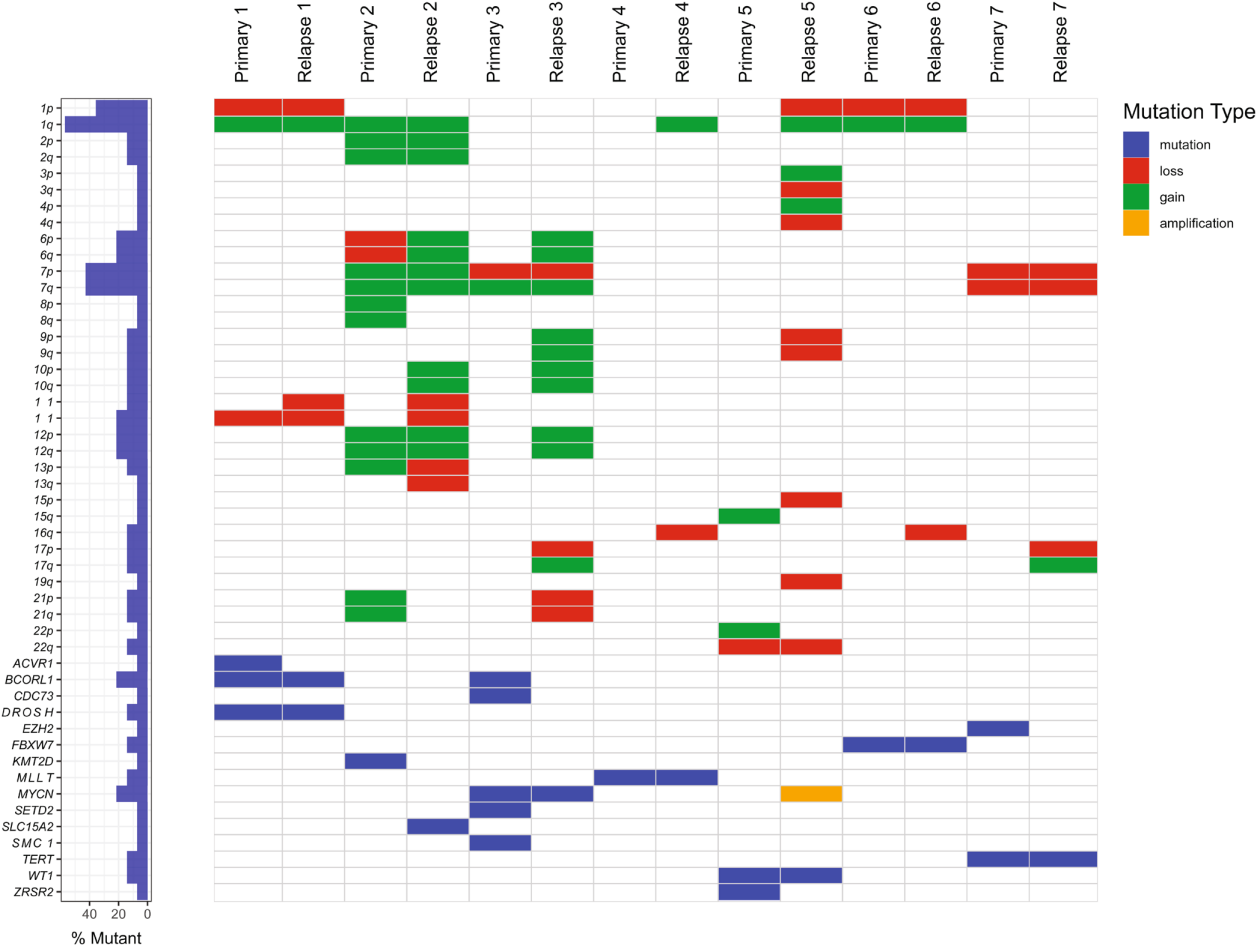
Copy number variations and mutated genes in paired primary and relapsed WT samples.

All primary and paired relapsed WTs underwent WXS, with the exception of primary case 4. The latter was sequenced using WGS. Based on WXS/WGS, mutations were identified in all WTs, including common (prevalence > 1%) WT drivers such as *WT1*, *MYCN*, *DROSHA*, and *BCORL1* (Table 3) [26]. Genes with mutations that were present in both primary and relapsed tumors of the same patient were considered to be clonal. These included *DROSHA*, *BCORL1*, *MYCN*, *MLLT1*, *WT1*, *FBXW7*, and *TERT*. Only one gene amplification (*MYCN*) was identified. CNVs observed in at least two relapses in our cohort included 1q gain (three primary samples and matched relapses, and two relapses (*de novo*)), 7q gain (two primary samples and matched relapses), as well as loss of 1p (two primary samples and matched relapses and one relapse (*de novo*)), 7p (two primary samples and matched relapses), 16q (two relapses (*de novo*)), and of 17p (two relapses (*de novo*)) (Figure 1). In one case (relapse 3), amplification of the *MYCN* locus (resulting from *NBAS‐MYCNOS* fusion) was a newly acquired aberration in the relapsed sample.

**TABLE 3:** Genetic aberrations in paired primary and relapsed WT.

Case	Primary			Relapse		
	Mutations/CNVs	Clonality	Fusions	Mutations/CNVs	Clonality	Fusions
1	*ACVR1 (2x)*	*Clonal*	None	*ACVR1 (2x)*	*Clonal*	None
	*DROSHA*	*Clonal*		*DROSHA*	*Clonal*	
	*BCORL1*	*Clonal*		*BCORL1*	*Clonal*	
	Loss of 1p, 11q			Loss 1p, 11q		
	Gain of 1q			Gain 1q		
				LOH chm 11p		
2	*KMT2D*	*Subclonal*	None	Gain of 1q		None
	Gain 1q			Gain of chrm 2, 6, 7, 10, 12		
	Gain chrm 2, 7, 8, 12, 13 and 21			LOH of chrm 11, 13		
	LOH chrm 6					
3	*MYCN*	*Clonal*	None	*MYCN*	*Clonal*	None
	*SMC1A*	*Clonal*		*SMC1A*	*Clonal*	
	*CDC73*	*Clonal*		*CDC73*	*Clonal*	
	*SETD2*	*Clonal*		*SETD2*	*Clonal*	
	*BCORL1*	*Clonal*		*BCORL1*	*Clonal*	
	Loss 7p			Gain of chrm 6, 9, 10 and 12		
	Gain 7q			Loss 7p and 17p		
				Gain 7q and 17q		
				LOH chrm 21		
4	*MLLT1*	*Clonal*	None	*MLLT1*	*Clonal*	None
				Gain 1q		
				Loss 16q		
5	*CDC73 (2x)*	*Clonal*	None	*CDC73 (2x)*	*Clonal*	*NBAS‐MYCNOS*
	*WT1*	*Clonal*		*WT1*	*Clonal*	
	*ZRSR2*	*Clonal*		*ZRSR2*	*Clonal*	
	Gain 15q, 22p			Amplification of *MYCN* locus		
	Loss 22q			Loss 1p, 3q, 4q, chrm 9, 15p, 19q, 22q		
				Gain 1q, 3p, 4p		
6	*FBXW7*	*Clonal*	None	*FBXW7*	*Clonal*	None
	Loss of 1p			Loss 1p, 16q		
	Gain of 1q			Gain 1q		
7	*TERT* promotor (c.‐146C>T)		None	*TERT* promotor (c.‐146C>T)		None
	*EZH2*			Loss chrm 7		
	Loss 7p, partial 7q			Loss 17p		
				Gain 17q		

Abbreviation: CNV, copy number variation.

Sequencing data on the control samples in our cohort were not available. For a general view of the (molecular) characteristics of an unbiased cohort of 30 primary WTs of patients treated in the Princess Máxima Center, we refer to van Belzen et al. [27].

#### 3.2.2 Differential Gene Expression Analysis: Primary WT Cases Versus Paired WT Relapses

**FIGURE 2:**
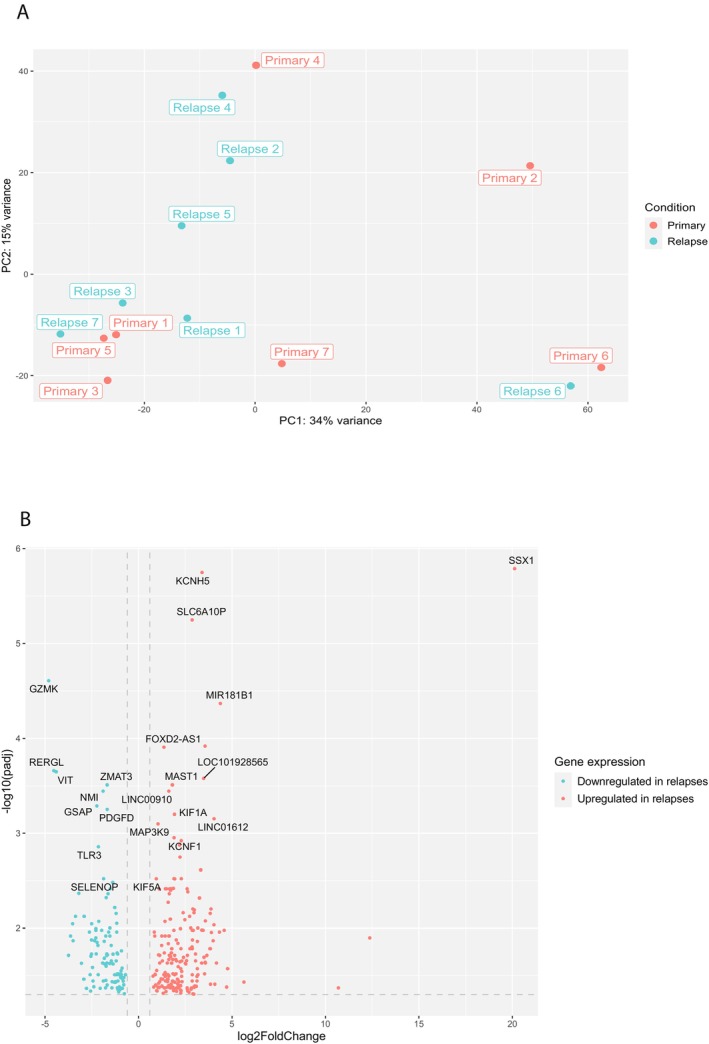
Differential gene expression comparing primary and paired relapsed WT samples. (A) Principal component analysis based on the top 500 genes (VST normalized). (B) Volcano plot showing all significantly differentially expressed genes. The most differentially expressed genes (based on adjusted *p*‐value) are annotated by gene symbol.

PCA was performed including the top 500 genes that showed the highest variability across the paired samples. The PCA showed that, in general, samples from the same patient clustered together (Figure 2A), except for sample 2 (intermediate‐risk WT). The top 50 genes that contributed to the variance of PC1 (which also explained most of the variance between primary and relapsed sample 2) are predominantly involved in immune processes. The top five significant Gene Ontology (GO) terms (biological processes) included: inflammatory response, immune effector process, regulation of immune system process, positive regulation of immune system process, and leukocyte mediated immunity. The top 50 genes are listed in Table S2, and all significant GO terms in Table S3.

**FIGURE 3:**
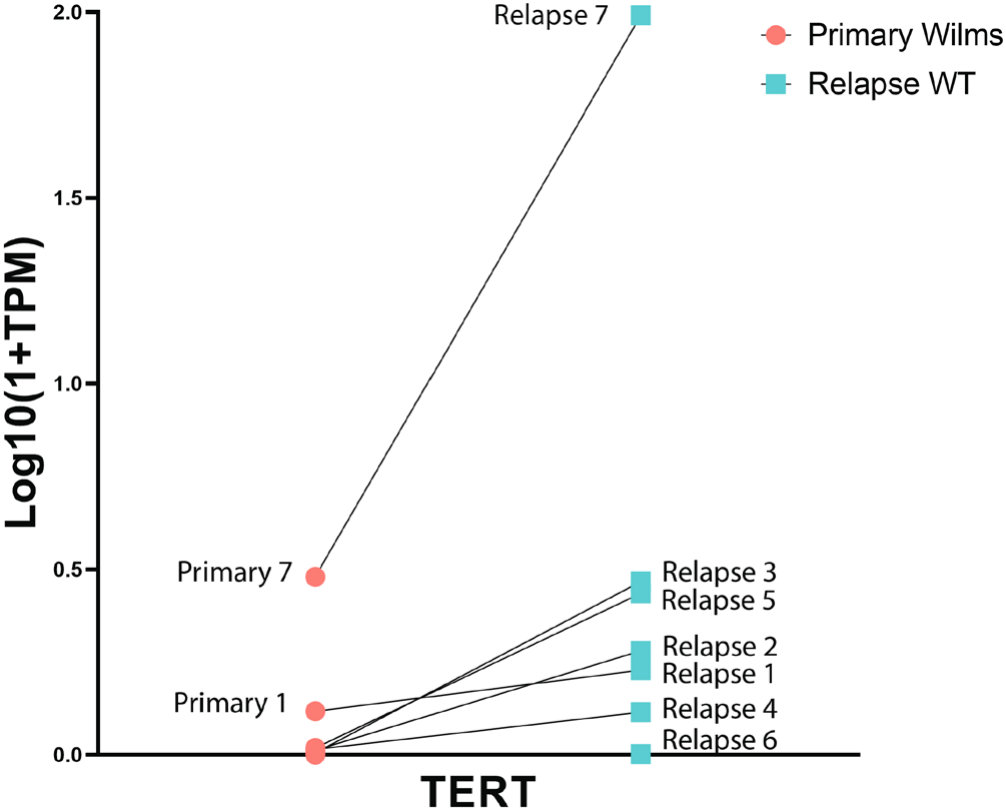
TERT expression (transcripts per million (TPM)) in primary and paired relapsed WT samples, compared using the Wilcoxon signed‐rank test. Patient 7 has a known TERT mutation (c.‐146C>T).

Differential gene expression analysis comparing the primary to the paired relapse samples resulted in 308 significantly differentially expressed genes, with 106 genes being downregulated in the relapsed samples compared to the primary samples, and 202 genes being upregulated in the relapsed samples compared to the primary samples (Figure 2B, and Supporting Information, File A). ToppFun analysis showed that the genes downregulated in relapsed samples correspond to gene sets involved in immune regulation, similar to the gene set explaining most of the variance in PC1. The top 15 significant GO terms (biological processes) are presented in Figure S1. The GO term analysis on the 202 genes that were upregulated in relapsed samples compared to the primary samples yielded four significantly enriched biological processes that were all related to dense core granules, which are commonly found in neurons and neuroendocrine cells (Figure S2) [28, 29]. This gene set was also enriched for genes related to stem cell regulation, based on the gene sets provided by the Progenitor Cell Biology Consortium [30]. The list with the gene set related to stem cell regulation is provided in Table S4. This list includes *TERT*, which has previously been described in relation to WT progression [11, 31, 32]. In our dataset, TERT expression was identified as significantly upregulated in the relapsed samples when compared to the primary WTs based on the VST normalized counts (Log2foldchange: 2.97 and adjusted *p*‐value 0.007), and comparison of the TPM counts of the paired samples (Wilcoxon signed‐rank test *p*‐value: 0.016). The change in *TERT* expression from primary to relapsed sample is depicted for each sample individually in Figure 3. Patient 7 had a *TERT* mutation (c.‐146C>T) both in the primary and relapsed tumor. This patient showed the highest expression of *TERT* both among the primary and relapsed samples, but not the steepest increase in expression levels when comparing the primary to the relapsed sample. The largest difference in expression levels between primary and relapsed sample was seen in patients 2 and 3. In addition, the upregulated genes (printed in bolt in Supporting Information, File A) were also associated with cerebral cortex based on the human protein atlas, and neuroepithelium based on the FaceBase [33] and the GenitoUrinary Development Molecular Anatomy Project (Gudmap) [34, 35]. It has previously been established that neural genes are associated with cancer stemness [36], including *NCAM* in WT stemness [37, 38, 39]. As further elaborated on in the discussion, both neural genes *PAX6* and *c‐KIT* have previously been found to be associated with WT [40]. Specifically, overexpression of c‐KIT has been shown to be associated with a worse prognosis in patients with WTs, including a shorter time to relapse [41, 42].

### 3.3 Identifying Expression Profiles Associated With (Risk of) Relapse

#### 3.3.1 Differential Gene Expression Analysis: Primary WT Cases That Later Relapsed Versus Primary WT Cases That Did Not Relapse (Controls)

**FIGURE 4:**
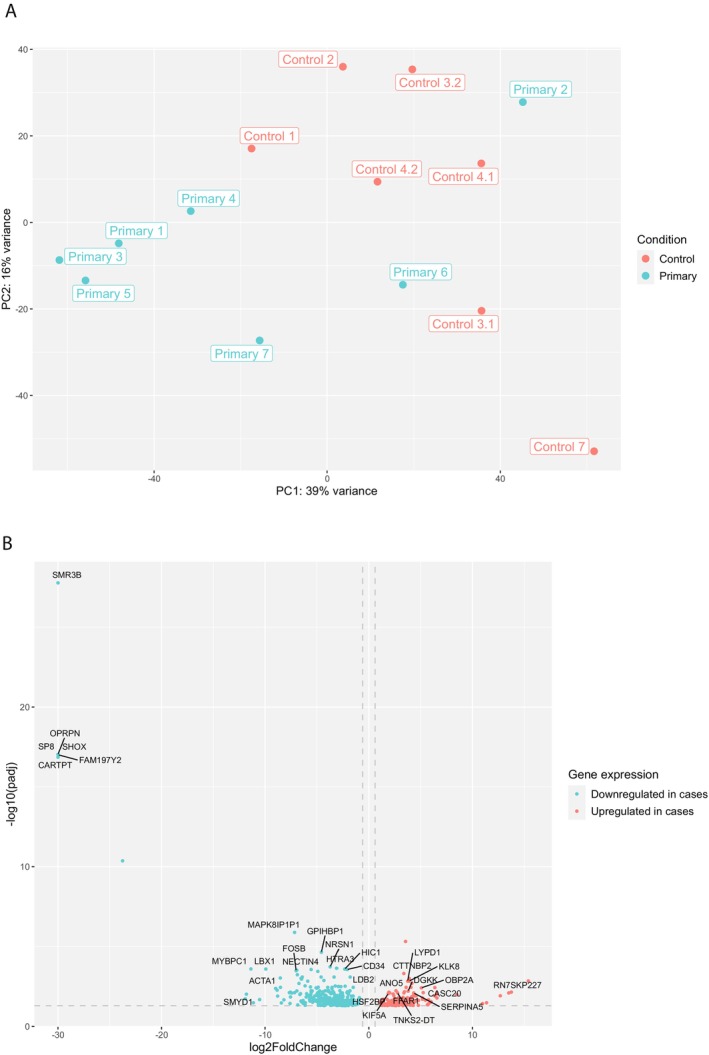
Differential gene expression comparing primary WT samples of patients with subsequent relapse (cases) and without (controls). (A) Principal component analysis based on the top 500 genes (VST normalized). (B) Volcano plot showing all significantly differentially expressed genes. The most differentially expressed genes (based on adjusted *p*‐value) are annotated by gene symbol.

To identify gene sets associated with (risk of) tumor relapse, we compared gene expression levels in the primary tumors of patients with relapse (cases) and matched patients without relapse (controls). PCA on the top 500 VST normalized gene counts showed clustering based on these two conditions (Figure 4A). Primary case 2, however, was an outlier and showed a gene expression pattern similar to that of control samples. Differential gene expression between primary tumors of patients with and without relapse identified 448 differentially expressed genes (*p*_adj_ < 0.05) (Figure 4B, and Supporting Information, File B). Out of the 448 differentially expressed genes, 303 genes were downregulated in cases compared to control samples, and 145 genes were upregulated. The downregulated genes were enriched for gene sets related to stromal cells, primarily based on gene sets of the Immunological Genome Project [43]. In addition, significant GO terms were associated with (skeletal) muscle (development), and the downregulated genes appeared to be significantly enriched for the skeletal muscle gene set provided by the human protein atlas. The upregulated genes in the primary tumors of the cases, when compared to the controls, appeared to be involved in stem cells based on the gene sets provided by the Progenitor Cell Biology Consortium. The gene set related to stem cell regulation is provided in Table S5. In addition, like the comparison between the primary and paired relapse samples, the upregulated genes also included neuro‐related genes. These were associated with cerebral cortex, brain tumors, and neuro‐epithelium based on the human protein atlas, The Cancer Genome Atlas (TCGA) and the gene set related to the human intestinal tract provided by Elmentaite et al. [44], respectively. Significant GO terms were associated with (transmembrane) peptide and ion transport, as well as carbohydrate homeostasis.

### 3.4 Immune Cell Deconvolution

**FIGURE 5:**
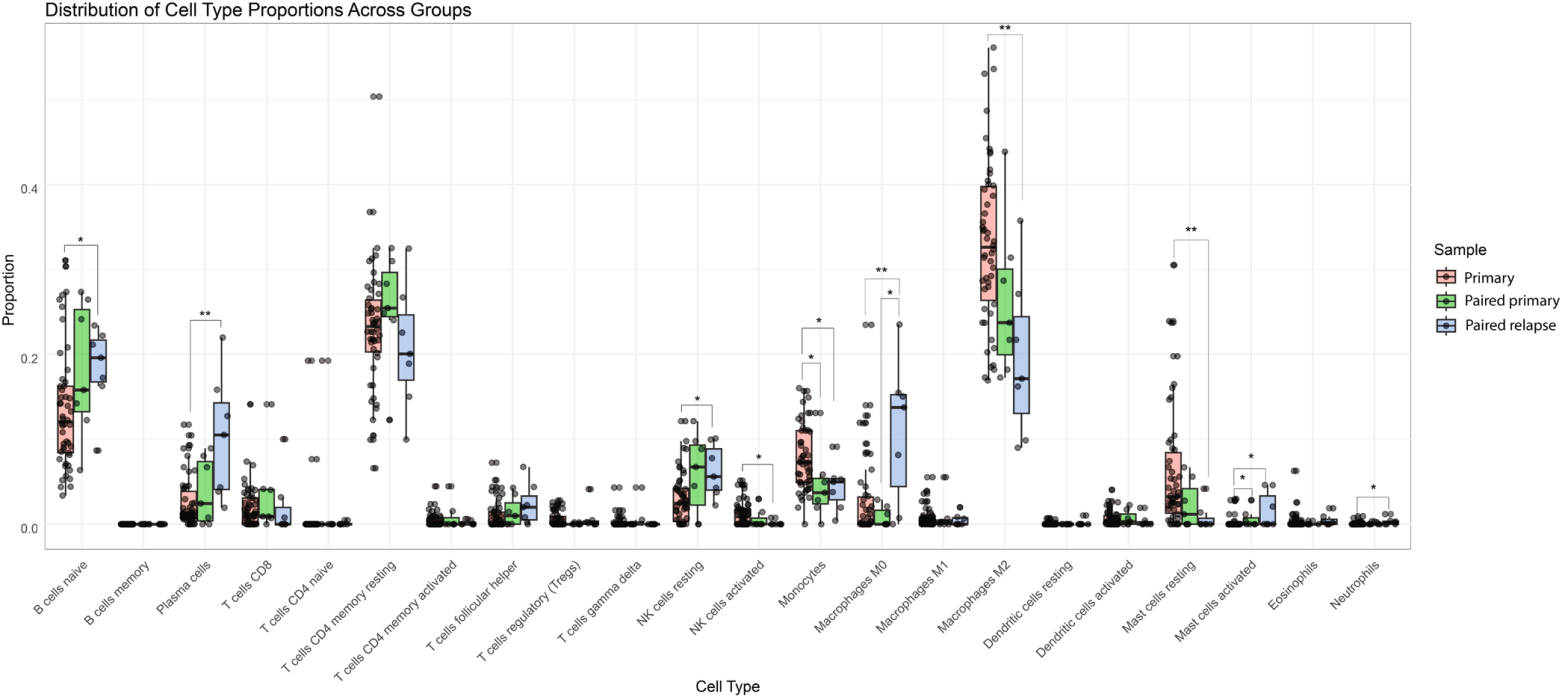
Distribution of immune cell type proportions across control samples and paired primary and relapse samples.

Paired sample analysis showed that gene sets involved in immune regulation were downregulated in relapsed WT. To further investigate which immune cells may specifically be involved in relapse, we performed CIBERSORTx immune cell deconvolution. The proportion of immune cells in primary samples and paired WT samples is presented in Figure 5. Only M0 macrophages were present in a significantly larger proportion in relapse samples than in paired primary samples. Although we did not observe differences in immune regulation between primary cases and matched controls in differential gene expression analysis, immune cell deconvolution did show a significantly smaller proportion of monocytes and a larger proportion of activated mast cells in paired primary cases compared to (all, including unmatched) unpaired primary samples. Moreover, comparing unpaired primary to relapsed WT samples, naïve B cells, plasma cells, resting NK cells, M0 macrophages, activated mast cells, and neutrophils were relatively more abundant in relapses. In contrast, proportions of activated NK cells, monocytes, M2 macrophages, and resting mast cells were larger in unpaired primary samples.

### 3.5 Relapse Prediction Model

**FIGURE 6:**
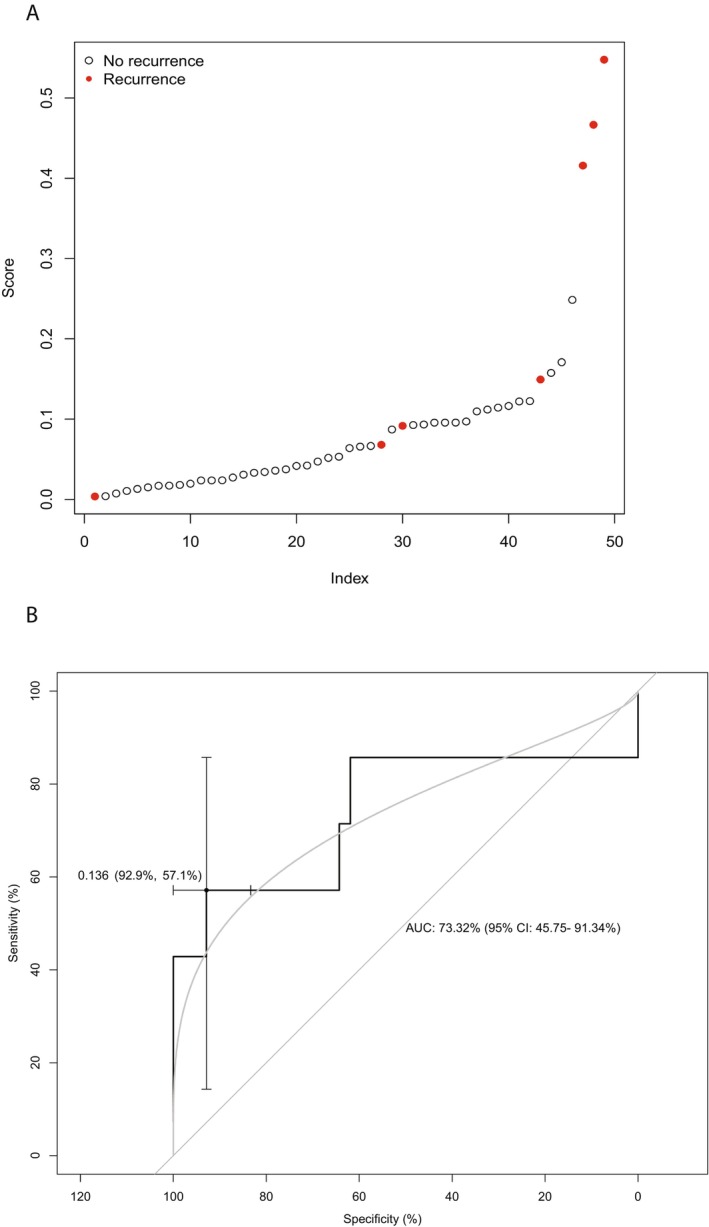
(A) Results of ridge regression WT relapse prediction model. (B) ROC‐curve with a maximized sensitivity and specificity at a threshold of 0.136.

Based on the gene expression of all primary WTs diagnosed in the Princess Máxima Center with relapse (*n* = 7) and without relapse (*n* = 42, characteristics described in Table S7), we constructed a ridge regression model for WT relapse using 10‐fold cross‐validation. The result of this model predicted the recurrences in each fold that was excluded (Figure 6A). The ROC plot derived from these results had an AUC of 74.15% (95% Confidence Interval (CI): 46.53%–100%) with an optimal threshold of 0.136, corresponding to a sensitivity of 57.14% (95% CI: 14.29%–85.71%) and a specificity of 92.86% (95% CI: 83.33%–100%) (Figure 6B). Smoothening the curve (bi‐normal (linear model)), represented by the gray curve in Figure 6B, resulted in an AUC of 73.32% (95% CI: 45.75%–91.34%), and optimal sensitivity and specificity of 54.60% and 84.47%, respectively. When comparing the characteristics of the patients (as presented in Table 2) that were correctly predicted to have a recurrence based on the threshold of 0.136, to the patients that were not recognized to have a recurrence (false negatives), we did not observe any significant differences, suggesting that the model did not specifically identify a known subset of relapses. The genes that had predictive value in at least eight of the 10 models (10‐fold cross‐validation, listed in Table S6) were run through ToppFun. GO term analysis showed that these genes were enriched for genes involved in (skeletal) muscle (development), similar to the differentially downregulated genes in cases compared to matched controls (Table S8). The model was not of prognostic value in the TARGET dataset as the predicted risk was not significantly associated with the time to relapse/progression in this cohort. The lack of validation may be due to the fact that the patients in the TARGET dataset were not pre‐treated with chemotherapy prior to nephrectomy.

## 4 Discussion

Fifteen percent of patients with a WT experience tumor recurrence. To unravel the molecular drivers of tumor recurrence, we first studied the gene mutation and expression profile of paired primary and relapsed WT samples. Mutational burden in all relapses was relatively low. In addition, our paired analysis suggests that immune dysregulation and high tumor stemness may drive tumor progression. Secondly, the comparison of gene expression data of primary WT cases with subsequent relapse and matched controls suggests that patients with reduced stromal differentiation and high stemness markers at primary diagnosis may be at increased risk of relapse. Using our regression model, these patients with an increased risk of relapse could be identified with a high specificity.

Firstly, we compared the gene mutation and expression profile of paired primary and relapsed WT samples. So far, few studies have addressed this issue previously. The Associazione Italiana Ematologia Oncologia Pediatrica (AIEOP) studied mutational status and CNVs in paired primary and relapse WT samples. Spreafico et al. identified chromosomal anomalies at 1q, 3, 16q, and mutations of *SIX1* and *DROSHA* as potential driving events in WT recurrence [9]. Ciceri et al., studying 27 paired samples, including the eight samples described by Spreafico et al., as well as 10 relapse samples without primary tissue available, corroborated the association between mutations in *SIX1/SIX2* and microRNA processing genes (miRNAPGs) and WT relapse [10]. The latest study by Ciceri et al. published in 2024 studied 24 paired samples, including 18 paired samples that had previously been described [13]. The authors, now applying a much larger gene panel of 5000 genes, identified clonal mutations *BLM, BMI1, BRCA2, CDC73, CHEK2, CREBBP, CTC1, CTNNB1, DICER1, DYNC1H1, FANCA, KMT5B, LTBP3, MUTYH, MYCN, PALB2, PAX8, PMS2, SDHB, SMAD3, SMARCAL1, TP53*, and *UBA1*. These genes were mostly involved in DNA damage prevention and repair or chromatin modification and regulation. Mutations in *SIX1* and miRNAPGs, as well as gain of chromosome 1q, were also found to be potential drivers of WT progression and relapse by the Children's Oncology Group Renal Tumor Committee (COG‐RTC) (*n* = 51 relapses (45 paired primary samples)) [11]. In addition, they identified recurrent mutations in genes involved in the MYCN network in relapsed WT. Another study by the COG‐RTC and United Kingdom Children's Cancer and Leukemia Group (UKCCLG) demonstrated that among 10 paired samples, chromosomal gains at 5p, 8p12, 15q, 16p, and 20q, and losses at 11q and 17p were acquired in more than one relapsed tumor [12].

In our study, the few mutations that were identified in the primary samples were maintained at relapse. It is well accepted that pediatric solid tumors have a low mutation burden [45]. In our paired samples, we observed mutations in established WT drivers [26, 46], including: *WT1*, *MYCN*, *DROSHA*, *BCORL1*, *TERT*, and *FBXW7*. An insertion in *MLLT1* was also maintained in the primary and relapsed tumor. Insertions in this region have previously been associated with primary and relapsed WT [11, 47]. Of the clonal mutations that we identified, *DROSHA* and *MYCN* had previously been described in the paired samples analyses by Spreafico et al. [9], and in the COG‐RTC [11]. As described by Ciceri et al., we too identified clonal mutations in *CDC73* [13]. We did not observe the association between SIX1/SIX2 mutations and WT relapse. This might be due to our relatively small sample size, as well as the fact that all our patients were pre‐operatively treated with chemotherapy. The studies by Spreafico and Ciceri et al. also included patients who underwent immediate nephrectomy, as is standard of care in the COG‐RTC [48]. The use of pre‐operative chemotherapy, in comparison to immediate nephrectomy, has previously been shown to significantly affect gene expression [49]. Although in different genes than presented by Ciceri et al., we too observed an abundance of clonally mutated genes involved in DNA repair and genome stability, as well as epigenetic regulation and chromatin remodeling (*EZH2* [50], *BCORL1* [51], *SETD2* [52], *SMC1A* [53], *CDC73* [54], *FBXW7* [55], *MLLT1* [47]).

Considering CNVs, it has been observed that genomic losses were more frequent in primary WTs of patients who would subsequently experience relapse [56]. In our paired primary and relapsed cases, the recurrent CNVs included gain of 1q and 7q, and loss of 1p, 7p, 16q, and 17p. Of these variations, anomalies at 1q, 16q, and loss of 17p had previously been reported in paired WT samples analysis by Spreafico et al. [9] and Natrajan et al. [12] Moreover, gain of chromosome 1q and loss of chromosomes 1p and/or 16q have previously been identified as adverse prognostic factors at primary diagnosis [57, 58], and the prevalence of 1q gain has been found to be higher in relapsed samples than (paired) primary cases [11, 56]. Gain of 7q has also previously been reported as an adverse factor [56], as has loss of 7p and *de novo* loss of 17p [9, 12, 56]. Krepisci et al. even pinpointed potential tumor suppressors located on 7p: *TWIST1* and *SOSTDC1*. Since *TP53* is located on 17p, loss of this chromosome arm is likely associated with anaplastic histology (and poor prognosis) [59, 60]. Interestingly, one sample with only one *MLLT1* mutation presented without any CNVs at primary diagnosis. At relapse, however, this patient showed both gain of 1q and loss of 16q (as well as the known *MLTT1* mutation). In line with previous observations that the number of CNVs is higher at relapse than at primary diagnosis, we show that this is also the case in all but one of our patients [12].

Paired sample analysis showed that gene sets involved in immune regulation are downregulated in relapsed WT. Byron et al. have shown that solid refractory and relapsed pediatric tumors are in general immune cold tumors. More specifically, the immune score found in WT samples (*n* = 5) was < 0.1, suggesting immune suppression in these tumors [61]. This immune suppressive microenvironment most likely facilitates outgrowth of tumor cells. From the CIBERSORTx analysis, we predicted more M0 macrophages in relapses compared to paired primary WT samples. M0 macrophages are considered to be in a resting state, without a specific function, and can polarize into M1 (pro‐inflammatory) macrophages and M2 (suppressive) macrophages [62]. Large proportions of M2 macrophages were previously found to be associated with poor OS in WT [63]. Additionally, monocytes were polarized to M2 macrophages when co‐cultured with WT cell [64]. In our cohort, the proportion of M2 macrophages was significantly lower in relapsed samples compared to controls. This finding is unexpected based on the literature and may be related to the fact that tumor‐associated macrophages are located in tumor stroma [63, 65].

When studying primary tumors, high numbers of cytotoxic (CD8+) tumor‐infiltrating lymphocytes at the tumor margins were associated with a decreased risk of tumor relapse [66]. We did not observe a significant difference in immune regulation between primary cases and matched controls, based on the expression profiles. Immune cell deconvolution did show a smaller proportion of monocytes and a larger proportion of activated mast cells in primary cases; the proportion of CD8^+^ T cells, however, was not significantly different. Studying spatial transcriptomics might give more insight into the immune status of the tumor margins in the primary tumors. Finally, activated NK cells have been shown to kill WT cells in blastemal and epithelial WT primary cultures [64]. A subset of cells (30%) in these cultures was resistant to NK‐mediated tumor cell killing. In our cohort, the proportion of activated NK cells was lower in relapsed samples compared to controls, while resting NK cells were more abundant in relapsed samples. Altogether, our findings suggest that there may be only limited room for immunotherapy in WT relapse treatment (as single agent). This could be reflected in the results of a phase 1 trial on ex vivo expanded multi‐tumor associated antigen specific cytotoxic T lymphocytes (MTAA‐CTLs) in 18 relapsed or refractory solid tumors [67]. This study included nine WTs (one adult), and among the seven evaluable WT patients, five patients ultimately had progressive disease, and the remaining two patients had stable disease.

In addition to differential immune regulation, the paired analysis showed upregulation of stem cell markers in relapsed WT cases based on the Progenitor Cell Biology Consortium, which founded a Central Cell Characterization Core and Bioinformatics Core to perform characterization of induced pluripotent stem cells [30]. In addition, the relapsed WT cases were enriched for neuro‐epithelial/neuroendocrine differentiation markers. The genes associated with neuro‐epithelium included *PAX6* and *c‐KIT*. *PAX6* is known for its role in aniridia. The mutation, combined with a WT1 mutation, is a hallmark in WAGR syndrome, known to cause aniridia and WTs. A rare case of an isolated *PAX6* mutation causing both aniridia and WT has previously been reported [40]. Overexpression of c‐KIT, although rare in WT, has been reported by others and is thought to relate to a worse prognosis (including a shorter time to relapse) [41, 42]. Considering a broader perspective, it has previously been established that neural genes are associated with cancer, that the default fate of embryonic pluripotent cells is neural, and that these neural progenitor‐like cells/neural stem‐like cells are tumorigenic and can differentiate into all germ layers [36, 68, 69, 70]. Cancer stem cells (CSCs) are considered epithelial progenitor cells with dedifferentiation capacity to form blastemal cells and early mesodermal cells [71]. It is suggested that CSCs are capable of driving WT development. NCAM‐expressing CSCs, which were shown to overexpress WT stemness and progenitor genes (e.g., *WT1, SIX2, EZH2, BMI‐1, FZD7, NANOG*) [37, 38], have been identified in WT [39]. When comparing clusters of CSCs (specifically CSCs positive for SIX2 and CITED1) in normal human fetal kidney to the CSCs in WT, WTs were highly enriched in pathways related to muscle differentiation and regulation of the immune system [72]. The authors of this paper conclude that the CSCs (nephrogenic progenitor‐like cells) in WT are either maintained in a self‐renewal state or geared towards differentiation other than nephrogenesis. Altogether, we observed a high degree of (neuronal) stemness in relapsed WT samples. The stemness phenotype has been associated with antitumor immune activity [73]. Moreover, WT CSCs specifically have been shown to influence immune regulation and, CSCs in general, can create an immunosuppressive microenvironment [74]. These observations may tie in with the reduced immune regulation that we observed among the (paired) relapsed cases, although we cannot confirm a causal relationship between stemness and immune regulation in our study. One specific CSC‐related gene with upregulated expression in the relapsed samples was *TERT*. This gene has previously been shown to be associated with WT progression [11, 31, 32]. We too showed increased expression levels of *TERT* in relapsed cases compared to paired primary tumors, as well as increased expression in case of mutant *TERT* compared to non‐mutant *TERT*.

Secondly, we compared the gene expression of primary WT cases with subsequent relapse to matched controls. In this comparison, the (neuronal) stem cell markers were found to be upregulated in cases as well. The CSCs that differentiate to mesodermal cells might underlie the occurrence of muscle and bone that are sometimes seen in WT [71]. In our study, the cases showed downregulation of stromal cells/(skeletal) muscle (differentiation) markers, and the genes that were included in the prediction model were enriched for genes involved in (skeletal) muscle (development). One might argue that differentiation of CSCs to mesenchyme reduces the pool of CSCs with self‐renewal capacities that can propagate the tumor bulk, and thus that mesenchymal differentiation may be associated with reduced risk of tumor relapse. This view is supported by the favorable outcome of patients with stromal‐type WTs in general [75]. Secondly, also in other WTs, the rhabdomyoblast/tumor mass ratio after chemotherapy and the presence of (*WT1* mutation‐associated) muscle differentiation seemed to be associated with better prognosis [76, 77, 78]. Interestingly, previous work by van Belzen et al. suggested four WT groups based on gene expression, including one group that showed enrichment for muscle differentiation (with GO terms that are shared with our dataset) [27]. Within this group, 1q gain was a recurrent CNV (three out of seven tumors), which is generally considered an adverse prognostic factor for tumor relapse [4, 57]. Unfortunately, the relapse status of the patients described by our colleagues was unknown. Therefore, it is uncertain if this group of patients with 1q gain has an increased risk of relapse despite the high expression of genes related to muscle differentiation. Future studies with more patients and associated outcome data are needed to further test this hypothesis.

Based on the transcriptome, we aimed to predict which patient at diagnosis might be at risk of relapse. Our regression model was built using cross‐validation and resulted in an ROC‐curve with an AUC of 73.32% (95% CI: 45.75%–91.34%). An AUC between 70%–80% is considered acceptable [79], and although sensitivity was rather low (57.14% (95% CI: 14.29%–85.71%)), a specificity of 92.86% (95% CI: 83.33%–100%) suggests that the model is unlikely to classify patients without actual risk of relapse as being at risk. Therefore, the results of the model may be used to select patients eligible for treatment intensification intended to reduce the risk of relapse, without overtreating patients who are less likely to relapse. Before clinical application, however, the model must be externally validated in a large set of WT patients. We did not succeed in validating the model in the TARGET WT dataset. It is conceivable that validation was not successful because the patients included in the TARGET dataset are patients treated by the COG, who advocate for immediate nephrectomy, rather than pre‐operative chemotherapy.

Limitations of the study include the small number of available (paired) relapsed samples. Yet, the patients included in this study represent a national cohort of relapsed WT patients of the last 5 years, diagnosed and treated in a national pediatric oncology center. Another limitation is that our findings are primarily based on gene list enrichment analysis. Consequently, causal relationships between up‐ and downregulated gene sets, or the mutational and copy number status, cannot be drawn. Functional validations are required to obtain a deeper understanding of the interplay between the involved biological mechanisms. Finally, although we included all nationally available patients with a WT in the prediction model, the number of patients was not high enough to externally validate the model, which is required before clinical application can be considered.

In summary, WT driving mutations were maintained in relapsed tumors, and only one *de novo* mutation was observed at relapse. The known recurrent CNVs were also present in our cohort. On the transcriptome level, the relapses (compared to paired primary samples) and the primary samples of patients with subsequent relapse (compared to primary samples of patients without relapse) both showed upregulation of stemness markers. The increased level of stemness may contribute to an immunosuppressive tumor microenvironment and propagation of the tumor bulk. As such, the stem cell pool likely plays an important role in tumor relapse, and further differentiation of the neural/CSCs into mesenchymal cells might attenuate the risk of relapse. Our regression model might aid in selecting patients with an increased risk of relapse at primary diagnosis. However, external validation is required.

## Author Contributions

Alissa Groenendijk, Annelies M.C. Mavinkurve‐Groothuis, Marry M. van den Heuvel‐Eibrink, Ronald R. de Krijger, and Lennart Kester performed study concept and design; Alissa Groenendijk, Ronald R. de Krijger, and Lennart Kester performed the development of methodology, analysis, interpretation of the data and writing, review, and revision of the paper; Jarno Drost, Annelies M.C. Mavinkurve‐Groothuis, Martine van Grotel, Geert O. Janssens, Annemieke S. Littooij, Alida F.W. van der Steeg, and Marry M. van den Heuvel‐Eibrink provided review and revision of the paper; all authors read and approved the final paper.

## Ethics Statement

The study was approved by the local Biobank and Data Access Committee (PMCLAB2022.0311), and all patients had given informed consent.

## Conflicts of Interest

The authors declare no conflicts of interest.

## Supporting information


Figures S1–S2.

Table S1.

Table S2.

Table S3.

Table S4.

Table S5.

Table S6.

Table S7.

Table S8.

**File A.** Results of differential gene expression analysis comparing the primary Wilms tumors to the paired relapse samples. Upregulated genes involved in cerebral cortex and neuro‐epithelium are highlighted in yellow.

**File B.** Results of differential gene expression analysis comparing primary Wilms tumors of patients with and without relapse.

## Data Availability

The data that support the findings of this study are openly available in European Genome‐Phenome Archive at https://ega‐archive.org/.
