# Supplementary material for: Gene Expression Analysis of (Paired) Primary and Relapsed Wilms Tumor Samples to Unravel the Underlying Factors Driving Tumor Recurrence
Source: Cancer Med. 2025 May 29;14(11):e70969. doi: 10.1002/cam4.70969 (PMC12120524; doi:10.1002/cam4.70969)
Supplement: Supplementary file 1 — Figures S1–S2. [file CAM4-14-e70969-s004.pdf]

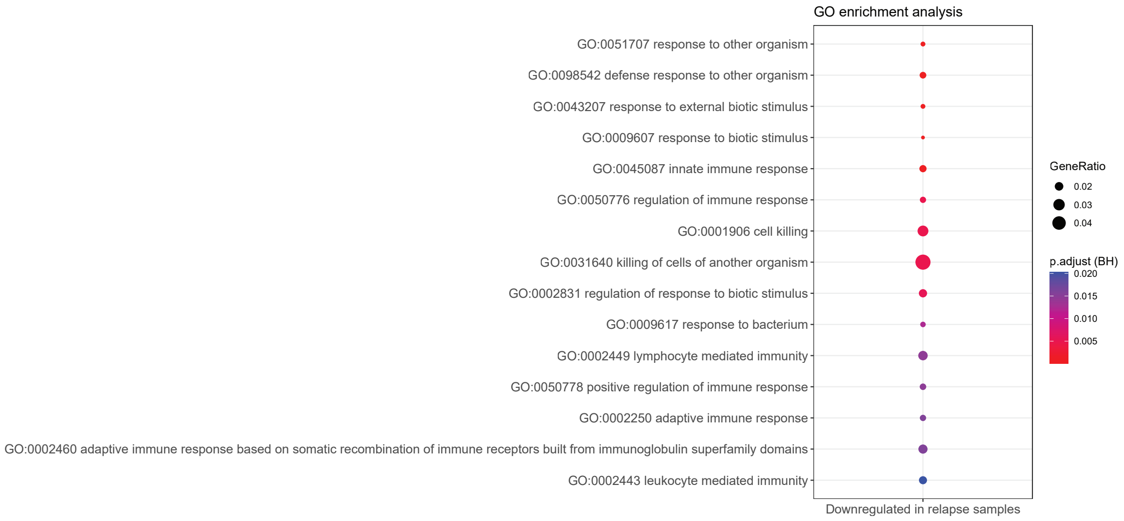

Supplementary Figure 1. Top 15 enriched Gene Ontology (GO) terms, including only biological processes, following GO term enrichment analysis on the genes that were significantly downregulated in relapse compared to primary samples.

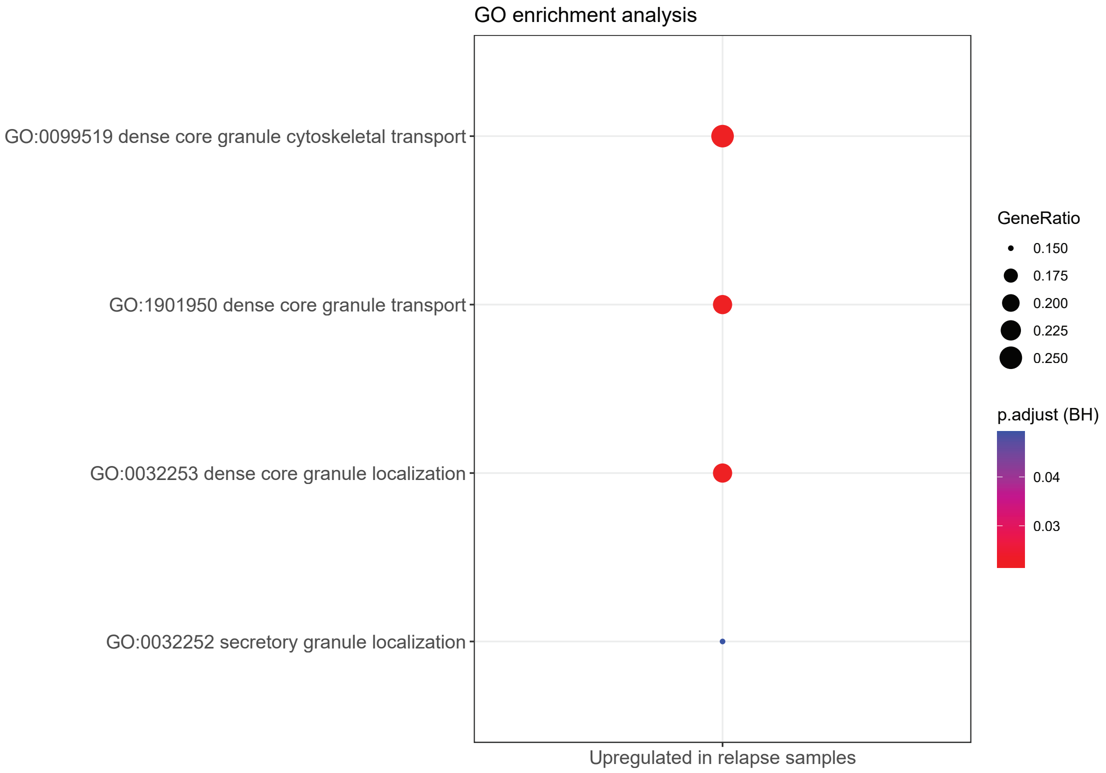

Supplementary Figure 2. Enriched Gene Ontology (GO) terms, including only biological processes, following GO term enrichment analysis on the genes that were significantly upregulated in relapse compared to primary samples.
